# Increased Brain Age Gap Estimate (BrainAGE) in Young Adults After Premature Birth

**DOI:** 10.3389/fnagi.2021.653365

**Published:** 2021-04-01

**Authors:** Dennis M. Hedderich, Aurore Menegaux, Benita Schmitz-Koep, Rachel Nuttall, Juliana Zimmermann, Sebastian C. Schneider, Josef G. Bäuml, Marcel Daamen, Henning Boecker, Marko Wilke, Claus Zimmer, Dieter Wolke, Peter Bartmann, Christian Sorg, Christian Gaser

**Affiliations:** ^1^Department of Neuroradiology, School of Medicine, Technical University of Munich, Munich, Germany; ^2^TUM-NIC Neuroimaging Center, School of Medicine, Technical University of Munich, Munich, Germany; ^3^Department of Anesthesiology, School of Medicine, Technical University of Munich, Munich, Germany; ^4^Functional Neuroimaging Group, Department of Diagnostic and Interventional Radiology, University Hospital Bonn, Bonn, Germany; ^5^Department of Neonatology, University Hospital Bonn, Venusberg-Campus, Bonn, Germany; ^6^Department of Pediatric Neurology and Developmental Medicine and Experimental Pediatric Neuroimaging group, University of Tübingen, Tübingen, Germany; ^7^Department of Psychology, University of Warwick, Coventry, United Kingdom; ^8^Warwick Medical School, University of Warwick, Coventry, United Kingdom; ^9^Department of Psychiatry, School of Medicine, Technical University of Munich, Munich, Germany; ^10^Department of Psychiatry, University Hospital Jena, Jena, Germany; ^11^Department of Neurology, University Hospital Jena, Jena, Germany

**Keywords:** magnetic resonance imaging, brain, development, aging, premature birth

## Abstract

Recent evidence suggests increased metabolic and physiologic aging rates in premature-born adults. While the lasting consequences of premature birth on human brain development are known, its impact on brain aging remains unclear. We addressed the question of whether premature birth impacts brain age gap estimates (BrainAGE) using an accurate and robust machine-learning framework based on structural MRI in a large cohort of young premature-born adults (*n* = 101) and full-term (FT) controls (*n* = 111). Study participants are part of a geographically defined population study of premature-born individuals, which have been followed longitudinally from birth until young adulthood. We investigated the association between BrainAGE scores and perinatal variables as well as with outcomes of physical (total intracranial volume, TIV) and cognitive development (full-scale IQ, FS-IQ). We found increased BrainAGE in premature-born adults [median (interquartile range) = 1.4 (−1.3–4.7 years)] compared to full-term controls (*p* = 0.002, Cohen’s *d* = 0.443), which was associated with low Gestational age (GA), low birth weight (BW), and increased neonatal treatment intensity but not with TIV or FS-IQ. In conclusion, results demonstrate elevated BrainAGE in premature-born adults, suggesting an increased risk for accelerated brain aging in human prematurity.

## Introduction

Premature birth, i.e., birth before 37 weeks of gestation, has a worldwide prevalence of around 11% (Chawanpaiboon et al., 2019). Survival rates of very (<32 weeks) and extremely (<28 weeks) premature-born individuals, in particular, have increased over the last decades (Howson et al., 2013; Glass et al., 2015). Prematurity has an impact on brain structure both on a microscopic and macroscopic level, establishing it as a paradigmatic neurodevelopmental disorder (Back et al., 2002; Nosarti et al., 2008; Deng, 2010; Buser et al., 2012; Ball et al., 2013; Dean et al., 2013; Volpe, 2019). These changes have been mostly found in children but there is increasing evidence for persistence of prematurity effects on the human brain until adulthood (Nosarti et al., 2008; Sølsnes et al., 2015; Zhang et al., 2015; Lefèvre et al., 2016). For example, aberrant gyrification and white matter alterations have been described in premature-born adults and were linked to functional deficits (Meng et al., 2016; Hedderich et al., 2019).

Brain aging has been studied extensively on a molecular and macroscopic level, mostly as a predisposing factor for neurodegenerative disorders late in life (Yankner et al., 2008). Examples for identified age-related brain alterations are decreased dendritic arborization, decreased neuronal plasticity, neuron cell loss, and decreasing white matter density (Bartzokis et al., 2003; Hedden and Gabrieli, 2004; Burke and Barnes, 2006; Yankner et al., 2008). Changes in gene expression patterns and defective DNA repair mechanisms have been proposed as putative underlying causes for age-related brain alterations (Lu et al., 2004; Fraser et al., 2005). However, the full picture of aging processes in the brain remains elusive to date. Recently, large-scale MRI studies have highlighted that aging effects are not limited to late-life but exist throughout the life course with spatially differential effects on gray matter and white matter (Coupé et al., 2017). Moreover, due to the increasingly recognized importance of early and mid-adulthood as a window for interventions aiming at the prevention of age-related brain disorders such as Alzheimer’s disease, the need for a valid biomarker of brain aging over the life span was emphasized (Belsky et al., 2015; Elliott et al., 2019). One such concept relies on the fact that some people experience faster biological degradation than others, resulting in an offset between “biological” and “chronological” age (Belsky et al., 2015; Elliott, 2020). Concerning the brain, this offset, or “brain age gap estimation” (BrainAGE) can be measured on structural MRI using machine-learning algorithms trained on large datasets (Franke et al., 2010; Cole and Franke, 2017; Franke and Gaser, 2019). This biomarker has proven sensitive to various neurological and neuropsychiatric conditions not only from the spectrum of dementing disorders in late-life but also in much younger patients with multiple sclerosis or schizophrenia (Kaufmann et al., 2019; Wang et al., 2019). Thus, it seems a promising biomarker candidate to find subtle manifestations of aberrant brain aging so early in life, when brain development and aging are still highly interrelated (Elliott et al., 2019). Recent evidence on metabolic and physiologic aging in premature-born adults in young adulthood suggests increased aging rates based on a previously established 10-item composite score (Belsky et al., 2015; Darlow et al., 2020). However, evidence about age-related, structural brain changes after premature birth is scarce and conflicting results exist, postulating both accelerated and delayed brain maturation (Franke et al., 2012; Karolis et al., 2017).

In the current study, we investigate the impact of premature birth on biological brain age in young adulthood in a large cohort of individuals born very preterm and/or at very low birth-weight (VP/VLBW) and age-matched controls born at full-term (FT), who were followed from birth until adulthood in a longitudinal, population-based cohort study. We address this question using structural MRI, a robust and accurate machine learning algorithm, and intelligence assessments at 26 years of age. Specifically, we hypothesized that BrainAGE will be altered in young adulthood and linked to perinatal variables of premature birth. Furthermore, we hypothesized that these alterations in BrainAGE are distinct from established markers of physical and cognitive developmental outcomes.

## Materials and Methods

### Participants

The participants examined in this study are part of the Bavarian Longitudinal Study (BLS), a geographically defined, whole-population sample of neonatal at-risk children and healthy full-term controls who were followed from birth into adulthood (Nosarti et al., 2008; Ball et al., 2012, 2014; Meng et al., 2016; Grothe et al., 2017). Of the initial 682 infants born very preterm (VP; <32 weeks) and/or with very low birth weight (VLBW; <1,500 g), 411 were eligible for the 26-year follow-up assessment, and of those, 260 (63.3%) participated in psychological assessments (Riegel et al., 1995; Wolke and Meyer, 1999). Of the initial 916 full-term born infants from the same obstetric hospitals that were alive at 6 years, 350 were randomly selected as control subjects within the stratification variables of sex and family socioeconomic status to be comparable with the VP/VLBW sample. Of those, 308 were eligible for the 26-year follow-up assessment, and 229 (74.4%) participated in psychological assessments. All of the 260 subjects from the VP/VLBW group underwent an initial screening for MR-related exclusion criteria, which included claustrophobia, inability to lie still for >30 min, unstable medical conditions (e.g., severe asthma), epilepsy, tinnitus, pregnancy, non-removable MR-incompatible metal implants and a history of severe CNS trauma or neurological disease. The most frequent reason not to perform the MRI exam, however, was lack of motivation. The remaining eligible, 101 VP/VLBW and 111 FT individuals underwent MRI at 26 years of age. The distribution of gestational age and birth weight in the VP/VLBW group is depicted in the supporting information (Supplementary Figure 1).

The MRI examinations took place at two sites: The Department of Neuroradiology, Klinikum rechts der Isar, Technische Universität München (*n* = 145), and the Department of Radiology, University Hospital of Bonn (*n* = 67). The study was carried out following the Declaration of Helsinki and was approved by the local institutional review boards. Written informed consent was obtained from all participants. All study participants received travel expenses and compensation for participation. A more detailed description of participants, including incidental brain MRI findings, can be found in previous publications (Breeman et al., 2015).

### Birth-Related Variables

Gestational age (GA) was estimated from maternal reports on the first day of the last menstrual period and serial ultrasounds during pregnancy. In cases where the two measures differed by more than 2 weeks, clinical assessment at birth with the Dubowitz method was applied (Bauml et al., 2015; Grothe et al., 2017). Maternal age, infant birth weight (BW), and duration of hospitalization were obtained from obstetric records. Family socioeconomic status (SES) was assessed through structured parental interviews within 10 days of childbirth. SES was computed as a weighted composite score based on the profession of the self-identified head of each family together with the highest educational qualification held by either parent (Dubowitz et al., 1970).

### Neurocognitive Assessment

At 26 years of age, study participants were assessed using an abbreviated version of the German Wechsler Adults Intelligence Scale, Third Edition (WAIS-III; Bauer, 1988): The assessment took place before and independent of the MRI scan and was carried out by trained psychologists who were blinded to group membership, resulting in a full-scale intelligence quotient (FS-IQ).

### MRI Data Acquisition

MRI examinations were performed at both sites on either a Philips Achieva 3T or a Philips Ingenia 3T system using 8-channel SENSE head-coils. Subject distribution among scanner was as follows: Bonn Achieva 3T: 5 VP/VLBW, 12 FT, Bonn Ingenia 3 T: 33 VP/VLBW, 17 FT, Munich Achieva 3T: 60 VP/VLBW, 65 FT, Munich Ingenia 3T: 3 VP/VLBW, 17 FT. To account for possible confounds by the scanner-specific differences, statistical analyses included categorical dummy regressors for scanner identity as covariates of no interest. Across all scanners, sequence parameters were kept identical. Scanners were checked regularly to provide optimal scanning conditions. MRI physicists at both sites regularly scanned imaging phantoms, to ensure within-scanner signal stability over time. Signal-to-noise ratio (SNR) was not significantly different between scanners [one-way ANOVA with factor “scanner-ID” (Bonn 1, Bonn 2, Munich 1, Munich 2); *F*_(3,182)_ = 1.84, *p* = 0.11]. The imaging protocol included a high-resolution T1-weighted, 3D-MPRAGE sequence (TI = 1,300 ms, TR = 7.7 ms, TE = 3.9 ms, flip angle 15°; the field of view: 256 mm × 256 mm) with a reconstructed isotropic voxel size of 1 mm^3^.

### Preprocessing of MRI Data and Data Reduction

We used a modified approach of our preprocessing as described previously (Franke et al., 2010). T1-weighted images were preprocessed using the CAT12 toolbox^1^ and the SPM12 software^2^, running under MATLAB^3^. All T1-weighted images were corrected for bias-field inhomogeneities, then segmented into gray matter (GM), white matter (WM), and cerebrospinal fluid (CSF) within the same generative model and spatially normalized using an affine registration (Ashburner and Friston, 2005). The segmentation procedure was further extended by accounting for partial volume effects and by applying adaptive maximum a posteriori estimations (Rajapakse et al., 1997; Tohka et al., 2004). Preprocessing further included smoothing with 4 mm full-width-at-half-maximum (FWHM) smoothing kernels. Data were further reduced by applying principal component analysis to reduce computational costs, to avoid severe overfitting, as well as to get a robust and widely applicable age estimation model, utilizing the “Matlab Toolbox for Dimensionality Reduction^4^”.

### BrainAGE Model Training Sample

To train the age estimation framework, we used MRI data of 648 healthy subjects (275 male) from the publicly accessible cohorts: fCONN^5^, NIH^6^, IXI^7^, and C-MIND^8^ aged 11–70 years [mean (SD) = 27.2 (12.0) years]. T1-weighted images were preprocessed using the same pipeline as described in the previous section.

### BrainAGE Framework

The BrainAGE framework utilizes a machine-learning pattern recognition method, namely relevance vector regression (RVR; Tipping, 2000, 2001). It was developed to model healthy brain development and aging and subsequently estimate individual brain ages based on T1-weighted images (Franke et al., 2010). As suggested previously, a linear kernel was chosen, since age estimation accuracy was shown not to improve when choosing non-linear kernels (Franke et al., 2010). Thus and in contrast to support vector machines, parameter optimization during the training procedure was not necessary. Within this study, the framework was applied using the linear combination of preprocessed GM and WM images. Since a leave-one-out approach is widely used in machine learning approaches and has been shown to provide a conservative estimate of a predictor’s true accuracy (Dosenbach et al., 2010), model training and individual brain age estimation were done using leave-one-out-loops (i.e., the preprocessed GM and WM images of all subjects, except one, was used for training). Subsequently, the brain age of the left-out subject was estimated. PCA was performed on the training sample and the estimated parameters were subsequently applied to the test subjects. In general, the age regression model is trained with chronological age and preprocessed whole-brain structural MRI data (as described above) of the training sample, resulting in a complex model of healthy brain development and aging. In other words, the algorithm uses those whole-brain MRI data from the training sample to extract the salient features of healthy brain development and aging. Additionally, voxel-specific weights are calculated that represent the importance of each voxel within the specified regression task (i.e., healthy brain aging). For an illustration of the most important features (i.e., the importance of voxel locations for regression with age) that were used by the RVR to model normal brain aging and more detailed information please refer to Franke et al. (2010). Subsequently, the brain age of a test subject can be estimated using the individual tissue-classified MRI data (as described above), aggregating the complex, multidimensional aging pattern across the whole brain into one single value. In other words, all the voxels of the test subject’s MRI data are weighted by applying the voxel-specific weighting matrix. Then, individual brain age is calculated by applying the regression pattern of healthy brain aging and aggregating all voxel-wise information across the whole brain. The difference between estimated and chronological age yields the individual brain age gap estimation (BrainAGE) score, with positive values indicating accelerated and negative values indicating decelerated structural brain aging. Consequently, the BrainAGE score directly quantifies the amount of acceleration or deceleration of brain aging. Recent work has demonstrated that this method provides reliable and stable estimates of BrainAGE at a mean absolute error of 3.322 years, rendering this framework superior to several recently introduced deep learning algorithms (Franke and Gaser, 2019). Specifically, the BrainAGE scores calculated from two shortly delayed scans on the same MRI scanner, as well as on separate 1.5T and 3.0T scanners, produced intraclass correlation coefficients (ICC) of 0.93 and 0.90, respectively (Franke et al., 2012). Within the current study, the BrainAGE framework was applied using the preprocessed GM and WM images and we corrected for the different scanner sites in our BrainAGE model. For training the model as well as for predicting individual brain ages, we used “The Spider^9^”, a freely available toolbox including several machine learning algorithms running in MATLAB.

### Statistical Analysis

Normal distribution of data was assessed using the Shapiro-Wilk test. Group difference of BrainAGE between VP/VLBW and FT individuals was assessed using the nonparametric Mann-Whitney-U test. Differences of clinical variables between VP/VLBW and FT individuals were tested using Chi^2^ tests (sex, SES) or two-sample *t*-tests (age, GA, BW, maternal age, FS-IQ). Correlations between variables of premature birth, BrainAGE, and adult FS-IQ were tested using Spearman’s rho, restricted to the VP/VLBW group. Nonparametric partial correlation analyses to calculate associations between total intracranial volume (TIV), FS-IQ, and BrainAGE, corrected for sex. Cohen’s d was calculated for group differences based on Mann-Whitney U-test (Lenhard and Lenhard, 2016). Statistical significance was set at *p* < 0.05. Statistical analyses were carried out using SPSS (version 25.0, IBM Corp.).

## Results

### Sample Characteristics

There were no significant group differences regarding age (i.e., mean age of 26 years) at scanning (*p* = 0.765), sex (*p* = 0.167), socio-economic status (SES; *p* = 0.760), and maternal age at birth (*p* = 0.889). By design, VP/VLBW individuals had significantly lower gestational age (GA; *p* < 0.001) and lower birth weight (BW; *p* < 0.001) and were hospitalized for a longer time after birth (*p* < 0.001). VP/VLBW subjects had significantly lower adult FS-IQ scores (*p* < 0.001). Please see Table 1 for details.

**Table 1:** Demographical, clinical, and cognitive data.

	VP/VLBW (*n* = 101)			FT (*n* = 111)			
	M	SD	Range	M	SD	Range	*p* value
Sex (male/female)	58/43			66/45			0.167
Age (years)	26.7	± 0.61	25.7–28.3	26.8	± 0.74	25.5–28.9	0.765
GA (weeks)	30.5	± 2.1	25–36	39.7	± 1.1	37–42	<0.001
BW (g)	1,325	± 313	630–2,070	3,398	± 444	2,120–4,670	<0.001
Hospitalization (days)	72.2	± 26.4	24–170	6.9	± 3.0	2–26	<0.001
SES^a^ (a.u.)	29/44/28		1–3	35/50/26		1–3	0.760
Maternal age (years)	29.5	± 4.8	16–41	29.4	± 5.2	18–42	0.889
Full-scale IQ^b^ (a.u.)	94.1	± 12.7	64–131	102.5	± 11.9	77–130	<0.001

*Statistical comparisons: sex, SES with χ^2^ statistics; age, GA, BW, Hospitalization, maternal age, IQ, with two-sample *t*-tests. Abbreviations: BW, birth weight; FT, full-term; GA, gestational age; IQ, intelligence quotient; M, Mean; maternal age, maternal age at birth; SD, standard deviation; SES, socioeconomic status at birth; VP/VLBW, very preterm and/or very low birth weight. ^a^1 = upper class, 2 = middle class, 3 = lower class. ^b^Data are based on 97 VP/VLBW and 108 FT-born individuals*.

### BrainAGE Is Increased for VP/VLBW Adults and Associated With Variables of Premature Birth

**Figure 1:**
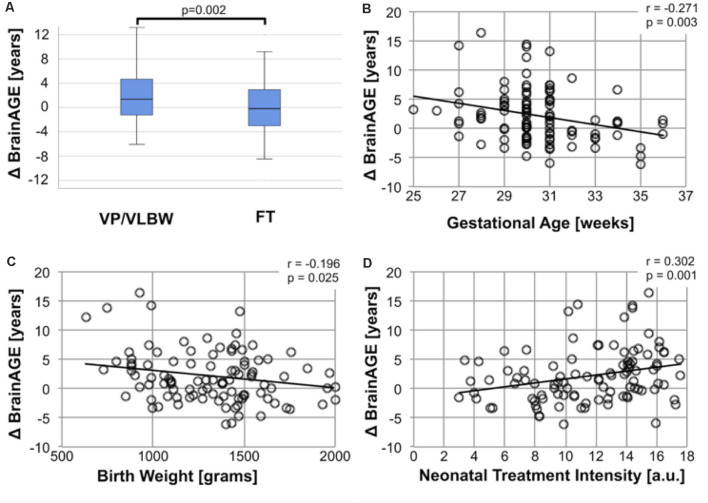
Increased BrainAGE after premature birth. **(A)** Group difference of BrainAGE between VP/VLBW and FT controls. Box plots of individual BrainAGE scores are shown for VP/VLBW and FT adults, indicating the median, interquartile range, and range. The median BrainAGE score is significantly elevated in VP/VLBW adults compared to FT controls as determined by Mann-Whitney U-test [VP/VLBW: 1.37 years, interquartile range (IQR): −1.26–4.67 years vs. FT: −0.19, IQR: −3.02–3.28 years; *p* = 0.002]. Increased BrainAGE is associated with perinatal variables of premature birth **(B–D)**. **(B)** ΔBrainAGE (y-axis) is plotted against gestational age (GA) at birth in weeks (x-axis) in VP/VLBW individuals. A linear regression line is added. Spearman’s r revealed a significant correlation (*r* = −0.271, *p* = 0.003). **(C)** ΔBrainAGE (y-axis) is plotted against birth weight (BW) in grams (x-axis) in VP/VLBW individuals. A linear regression line is added. Spearman’s r revealed a significant correlation (*r* = −0.196, *p* = 0.025). **(D)** Association between BrainAGE and neonatal treatment intensity. ΔBrainAGE (y-axis) is plotted against neonatal treatment intensity as measured by INTI (index of neonatal treatment intensity; x-axis) in VP/VLBW individuals. A linear regression line is added. Spearman’s r revealed a significant correlation (*r* = −0.302, *p* = 0.001). Abbreviations: BrainAGE, Brain Age Gap Estimate; FT, full-term; IQR, interquartile range; VP/VLBW, very preterm and/or very low birth weight.

To investigate the effect of premature birth on the difference between chronological age and biological brain age, we determined BrainAGE separately for premature-born adults (median = 1.37 years, interquartile range (IQR): −1.26–4.67 years) and full-term controls (median = −0.19, IQR: −3.02–3.28 years; Figure 1A). The difference between groups was statistically significant (*p* = 0.002) at a moderate effect size (Cohen’s *d* = 0.443). We interpret this result as increased biological brain age in premature-born adults. To test the specificity of this finding for premature birth, we ran Spearman correlation analyses between BrainAGE and variables of premature birth. We found significant correlations between BrainAGE and GA (*r* = −0.271; *p* = 0.003), BW (*r* = −0.196; *p* = 0.025) and intensity of neonatal treatment (INTI; *r* = 0.302; *p* = 0.001), underscoring that elevated biological brain age is specific for prematurity at birth (Figures 1B–D).

### BrainAGE Is Not Associated With Developmental Outcomes in Premature-Born Individuals

**Figure 2:**
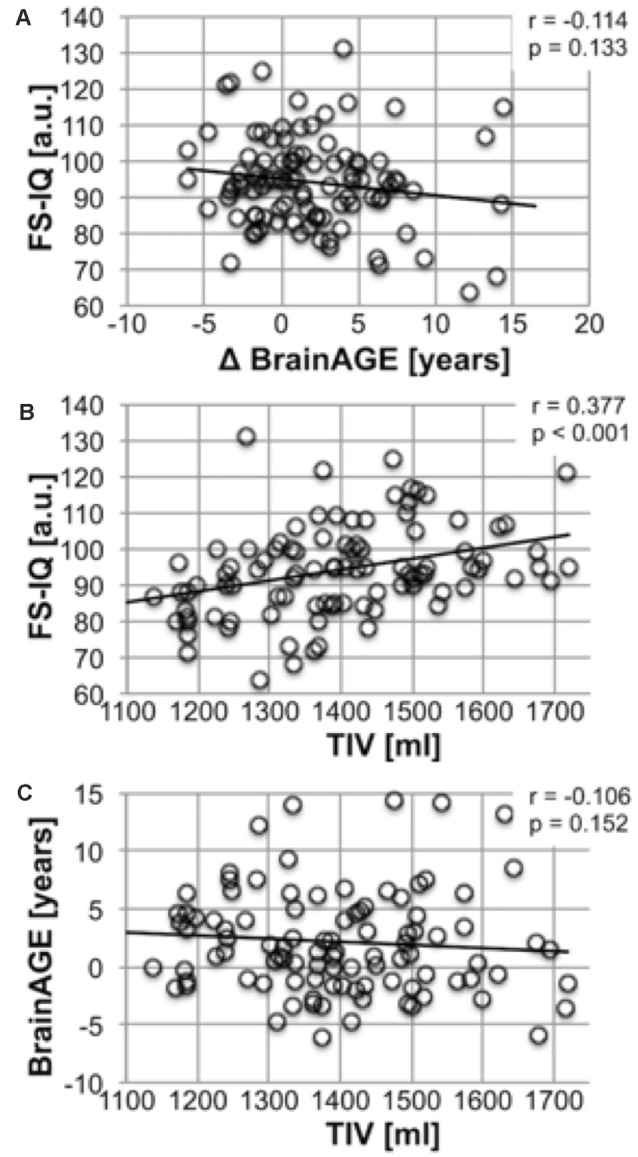
BrainAGE and variables of physical and cognitive outcomes after premature birth. Association between Full-scale IQ and BrainAGE. **(A)** Full-scale IQ (y-axis) is plotted against ΔBrainAGE in years (x-axis) in VP/VLBW individuals. A linear regression line is added. Joint partial correlation analysis with TIV corrected for sex revealed no significant correlation (*r* = −0.114, *p* = 0.133). Association between Total Intracranial Volume and Full-scale **(B)**. Full-scale IQ (y-axis) is plotted against TIV in ml (x-axis) in VP/VLBW individuals. A linear regression line is added. Joint partial correlation analysis with BrainAGE corrected for sex revealed a significant correlation (*r* = 0.377, *p* < 0.001). Association between TIV and BrainAGE **(C)**. BrainAGE in years (y-axis) is plotted against TIV in ml (x-axis) in VP/VLBW individuals. A linear regression line is added. Joint partial correlation analysis with FS-IQ corrected for sex revealed no significant correlation (*r* = −0.106, *p* = 0.152). Abbreviations: BrainAGE, Brain Age Gap Estimate; FS-IQ, full-scale intelligence quotient; FT, full-term; SD, standard deviation; TIV, total intracranial volume; VP/VLBW, very preterm and/or very low birth weight.

To investigate whether elevated BrainAGE is explained by altered developmental outcomes of cognitive and physical development, we analyzed the association between BrainAGE, TIV, and FS-IQ using non-parametric partial correlation analyses. Physical (TIV) and cognitive (FS-IQ) developmental outcomes were correlated significantly (*r* = 0.377; *p* < 0.001). No correlation was observed between BrainAGE and FS-IQ and TIV (*r* = −0.114; *p* = 0.133 and *r* = −0.106; *p* = 0.152; see Figure 2). We interpret the missing correlation between BrainAGE and developmental outcomes as support for our hypothesis that elevated BrainAGE reflects subtle manifestations of accelerated aging in early adulthood, which are not confounded by physical or cognitive-developmental outcomes.

## Discussion

Using structural MRI and a well-established machine-learning method for MRI-based estimation of the gap between chronological and biological brain age “BrainAGE”, we demonstrate significantly increased BrainAGE of approximately 1.4 years in premature-born adults at 26 years. We found this BrainAGE increase to be specific for premature birth through its associations with perinatal variables, namely low GA, low BW, and high neonatal treatment intensity, while links between BrainAGE and outcomes of physical and cognitive development were not found. Thus, our study adds to the knowledge about the long-term consequences of premature birth extending into the third decade of life and provides evidence for accelerated brain aging in premature-born adults.

The research about MRI-derived measurements of brain aging is still in its infancy and there exist mainly two distinct but not mutually exclusive frameworks that seek to explain the basis for increased BrainAGE (Elliott et al., 2019). A “geroscience” perspective argues that aging leads to the multi-organ accumulation and defective repair of damage on various levels, which is the basis for developing several diseases such as neurodegenerative disorders. Thus, this concept links aging with chronic diseases in late life, and an increase in BrainAGE would be interpreted as accelerated aging (Kennedy et al., 2014). The second perspective focuses on factors of imbalanced early system integrity and interprets an increase of BrainAGE as an indicator of compromised life-long brain health (Deary, 2012). In other words, a discrepancy between chronological and biological age may exist over a long period of life and may not change dynamically in terms of accelerated aging.

In the case of survivors of premature birth, it seems likely that early and persisting compromised system integrity plays a role throughout the life course affecting both the brain and the body. Premature birth is a risk factor for unfavorable cognitive development and several neuropsychiatric conditions such as attention deficit hyperactivity disorder (Nosarti et al., 2012; D’Onofrio et al., 2013; Wolke et al., 2019). But also organ function may be compromised affecting the lungs, the cardiovascular system, or the kidneys (Raju et al., 2017).

However, as stated earlier, this does not exclude a contribution of the geroscience perspective to our results. A recent epidemiologic study has found that BrainAGE was related to a “pace of aging” measure based on a composite score indexing multi-organ integrity in mid-life, thus underlining the possible interpretation of “accelerated aging” (Elliott et al., 2019). While a relationship between increased BrainAGE and the risk for neurodegenerative disorder has been established, it is still speculative in younger ages (Wang et al., 2019; Elliott, 2020). However, there is a biological rationale for this relationship since tau protein aggregation in neurons usually starts in peripheral dendrites and thus may reach the cell body faster in case of underdeveloped dendritic arborization as in premature-born humans (Dean et al., 2013; Back and Miller, 2014; Braak and Tredici, 2018). Moreover, neuropsychological evidence comes from a Finnish study that identified late premature birth as a risk factor for poor cognitive performance at 68 years of age (Heinonen et al., 2015). Although evidence on the relationship between premature birth and neurodegenerative disorders can only be regarded as preliminary, it constitutes an important question since after premature birth there is a whole life span available for potential preventive strategies.

While most studies on premature-born individuals use endpoints of successful development in early life, our results may motivate a life span perspective on survivors of premature birth (Wolke et al., 2019). Against the backdrop of cognitive decline in later life, improving cognitive abilities or educational attainment in the early development of premature-born individuals can also be viewed as a means to increase cognitive reserve throughout the life span (Lövdén et al., 2020). Apart from increasing the cognitive reserve through improving development and education, risk factor management in early adulthood may play an important role in the prevention of neurodegenerative disorders in later life (Belsky et al., 2015; Crous-Bou et al., 2017; Elliott et al., 2019). For example, modifiable risk factors for Alzheimer’s disease are either related to cardiovascular health or lifestyle habits (Crous-Bou et al., 2017). Interestingly, premature-born individuals bear a greater risk for hypertension, insulin resistance, and cardiovascular disease (Lawlor et al., 2005; Parkinson et al., 2013; Bavineni et al., 2019). Interventions aiming at successful risk factor management and promoting a healthy lifestyle may thus be of special importance for premature-born individuals as a particularly vulnerable group.

Importantly, our findings are limited by the cross-sectional study design and future studies with longitudinal follow-up of premature-born individuals well until mid-life and multiple time points of brain imaging will be needed in the future to assess BrainAGE changes over time. Also, while we have used FS-IQ as an overall measurement of cognitive performance and did not find an association with BrainAGE, testing of specific cognitive capacities over time may lead to a more comprehensive characterization of cognitive abilities of premature-born individuals in the context of aging.

In conclusion, we have shown specifically elevated BrainAGE scores in young adults after premature birth using a robust and well-validated machine-learning method. In line with the current literature, we hypothesize that accelerated brain aging processes contribute to this finding. Further research will need to address the relationship between premature birth and aging, particularly in long-term longitudinal studies on the effects of premature birth throughout the life course to develop biomarkers for early identification of at-risk populations for age-related brain disorders.

## Data Availability Statement

The datasets presented in this article are not readily available because of lacking consent of the participants. The original data is stored by the principal investigators of the Bavarian Longitudinal Study. Requests to access the datasets should be directed to the corresponding author.

## Ethics Statement

The studies involving human participants were reviewed and approved by Ethics committee University of Bonn, Medical Faculty and ethics committee Technical University of Munich, Medical Faculty. The participants provided their written informed consent to participate in this study.

## Author Contributions

DMH, HB, MW, CZ, DW, PB, CS, and CG designed the experiment. DMH, AM, BS-K, JGB, RN, JZ, SCS, and CG carried it out. DMH, AM, BS-K, MD, CS, and CG analyzed the data. DMH, PB, CS, and CG wrote the manuscript. AM, BS-K, RN, JZ, SCS, JGB, MD, HB, MW, CZ, and DW edited the manuscript. PB, DW, MW, CZ, CS, and CG supervised the work. All authors discussed the results and reviewed the manuscript. All authors contributed to the article and approved the submitted version.

## Conflict of Interest

The authors declare that the research was conducted in the absence of any commercial or financial relationships that could be construed as a potential conflict of interest.

## Footnotes

^1^http://www.neuro.uni-jena.de/cat

^2^http://www.fil.ion.ucl.ac.uk/spm/software/spm12

^3^http://www.mathworks.com

^4^http://lvdmaaten.github.io/drtoolbox

^5^http://www.nitrc.org/ir/app/action/ProjectDownloadAction/project/fcon_1000

^6^http://pediatricmri.nih.gov/nihpd/info/index.html

^7^http://brain-development.org/ixi-dataset/

^8^http://research.cchmc.org/c-mind/

^9^http://www.kyb.mpg.de/bs/people/spider/main.html

## Abbreviations

ANOVA: analysis of variance
BLS: Bavarian Longitudinal Study
BrainAGE: Brain Age Gap Estimate
BW: birth weight
FS-IQ: full-scale IQ
FT: full-term
FWHM: full-width at half maximum
GA: gestational age
INTI: intensity of neonatal treatment index
MPRAGE: magnetization prepared rapid acquisition gradient echo
MRI: magnetic resonance imaging
SES: socioeconomic status
TE: time to echo
TIV: total intracranial volume
TI: time to inversion
TR: time to repetition
VLBW: very low birth weight
VP: very preterm
WAIS: Wechsler Adult Intelligence Scale.
