# Supplementary material for: Increased Brain Age Gap Estimate (BrainAGE) in Young Adults After Premature Birth
Source: Front Aging Neurosci. 2021 Apr 1;13:653365. doi: 10.3389/fnagi.2021.653365 (PMC8047054; doi:10.3389/fnagi.2021.653365)
Supplement: Supplementary file 1 [file Data_Sheet_1.docx]

Supporting information for “Increased Brain Age Gap Estimate (BrainAGE) in Young Adults after Premature Birth” by Hedderich et al.

*Figure S.1: Scatter-plot showing the distribution of gestational age and birth weight in the premature-born adults cohort.*


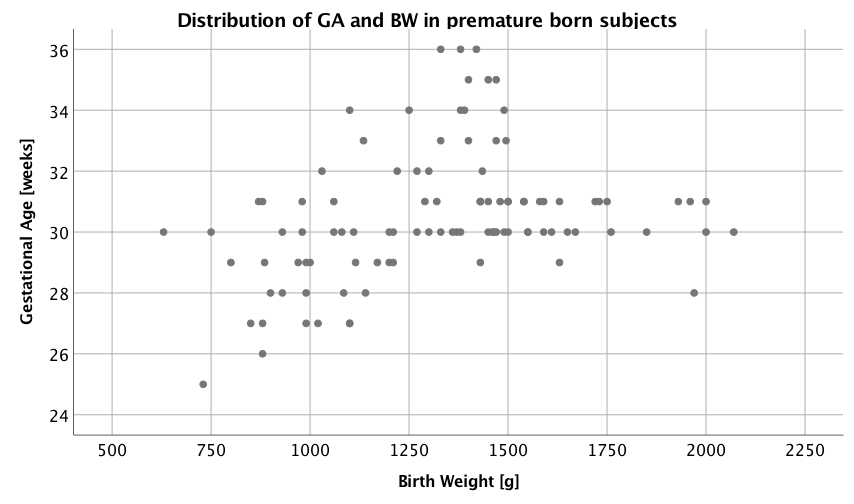


Abbreviations: GA: gestational age; BW: birth weight; g: grams
